# Calycosin-7-O-β-Glucoside Isolated from *Astragalus membranaceus* Promotes Osteogenesis and Mineralization in Human Mesenchymal Stem Cells

**DOI:** 10.3390/ijms222111362

**Published:** 2021-10-21

**Authors:** Kyung-Ran Park, Ji Eun Park, Bomi Kim, Il Keun Kwon, Jin Tae Hong, Hyung-Mun Yun

**Affiliations:** 1 Department of Oral and Maxillofacial Pathology, School of Dentistry, Kyung Hee University, Seoul 02447, Korea; rudfks282@naver.com; 2 Medical Device Research Center, Medical Science Research Institute, Kyung Hee University Medical Center, Seoul 02447, Korea; kwoni@khu.ac.kr; 3 National Institute for Korean Medicine Development, Gyeongsan 38540, Korea; chool9090@nikom.or.kr (J.E.P.); BomiKim@nikom.or.kr (B.K.); 4 Department of Dental Materials, School of Dentistry, Kyung Hee University, Seoul 02447, Korea; 5 College of Pharmacy and Medical Research Center, Chungbuk National University, Cheongju-si 28160, Korea

**Keywords:** AKT, ALP, BMP2, Caly, mineralization, MSCs, osteogenesis, RUNX2

## Abstract

Stem cells have received attention in various diseases, such as inflammatory, cancer, and bone diseases. Mesenchymal stem cells (MSCs) are multipotent stem cells that are critical for forming and repairing bone tissues. Herein, we isolated calycosin-7-O-β-glucoside (Caly) from the roots of *Astragalus membranaceus*, which is one of the most famous medicinal herbs, and investigated the osteogenic activities of Caly in MSCs. Caly did not affect cytotoxicity against MSCs, whereas Caly enhanced cell migration during the osteogenesis of MSCs. Caly increased the expression and enzymatic activities of ALP and the formation of mineralized nodules during the osteogenesis of MSCs. The osteogenesis and bone-forming activities of Caly are mediated by bone morphogenetic protein 2 (BMP2), phospho-Smad1/5/8, Wnt3a, phospho-GSK3β, and phospho-AKT, inducing the expression of runt-related transcription factor 2 (RUNX2). In addition, Caly-mediated osteogenesis and RUNX2 expression were attenuated by noggin and wortmannin. Moreover, the effects were validated in pre-osteoblasts committed to the osteoblast lineages from MSCs. Overall, our results provide novel evidence that Caly stimulates osteoblast lineage commitment of MSCs by triggering RUNX2 expression, suggesting Caly as a potential anabolic drug to prevent bone diseases.

## 1. Introduction

Mesenchymal stem cells (MSCs) are adult stem cells that are found in tissues including bone marrow, fat, and dental pulp [1]. MSCs are also multipotent stem cells that are capable of self-renewing and differentiating into diverse cell types through symmetric and asymmetric cell divisions, which have become a major source of stem cell therapeutics [2]. MSCs are physiologically and pathologically important cells for forming, remodeling, and repairing bone tissues [1]. In osteogenic conditions, human MSCs are committed to the osteoblast lineages that are the major cellular component of bone tissues, and the osteogenesis of MSCs leads to increases in alkaline phosphatase (ALP) activities and the mineralization of the bone matrix with apatite crystals [3]. The osteogenesis of MSCs is mainly mediated by BMP2 and Wnt3a, and their intracellular signaling molecules that regulate runt-related transcription factor 2 (RUNX2) expression [3]. Therefore, these osteogenic factors and signaling pathways are important targets for the treatment of bone diseases, such as osteoporosis and periodontitis, which are the main hallmarks of osteoblast dysfunction, including proliferation, migration, differentiation, and mineralization.

*Astragalus membranaceus,* known as Huangqi, is one of the oldest and most frequently used herbs for oriental medicine in Asian countries, including Korea, China, and Japan [4,5]. *A. membranaceus* possesses a variety of pharmaceutical properties and contains bioactive compounds to improve overall health and to prevent and cure disease including neurodegenerative diseases, diabetes, and cancers [6]. The main active compounds of *A. membranaceus* include polysaccharides, saponins, and flavonoids [7,8,9]. It was reported that Astragaloside I from *A. membranaceus* enhances osteoblast differentiation via Wnt/β-catenin signaling [10]. More recently, Astragaloside IV from *A. membranaceus* increases osteogenesis and alleviates osteoporosis via vitamin D/FGF23/Klotho signaling [11]. A bioactive compound, calycosin-7-O-β-glucoside (Caly), is an isoflavane belonging to flavonoids in *A. membranaceus* [6]. To date, the molecular mechanisms of Caly in human MSCs have not yet been reported.

In the present study, the bioactive compound Caly was obtained from the roots of *A. membranaceus*, and the beneficial effects of Caly on ostogenesis and bone-forming activities were investigated in human MSCs. 

## 2. Results

### 2.1. Isolation of Bioactive Compound Caly from the Roots of A. membranaceus and Its Effects on Cytotoxicity against Human MSCs

**Figure 1:**
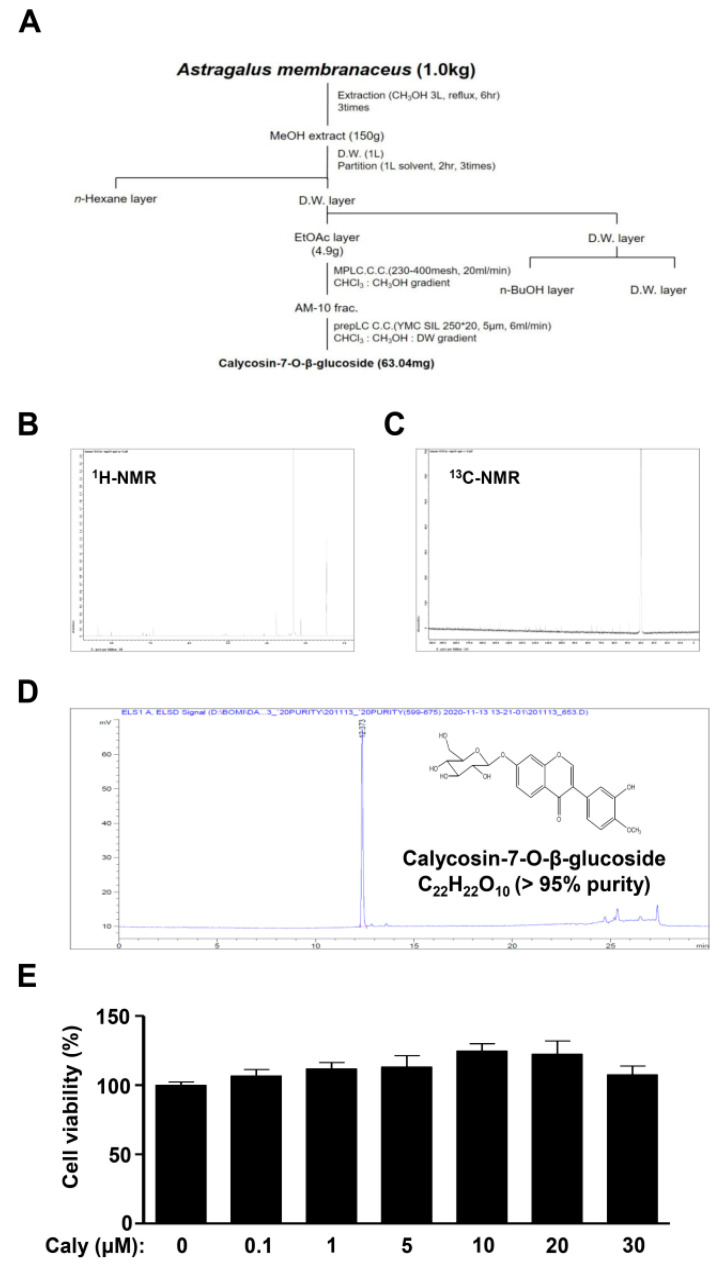
Isolation of Caly from the roots of *A. membranaceus*, and its effects on cytotoxicity against MSCs. (**A**) Roadmap of Caly isolated from root of *A. membranaceus*. (**B**,**C**) ^1^H and ^13^C NMR of Caly. (**D**) HPLC and chemical structure of Caly. (**E**) Caly (0.1, 1, 5, 10, 20, and 30 μM) was treated for 24 h in MSCs, and cell viability was analyzed using an MTT assay. Data are representative of three separate experiments.

*A. membranaceus* (1 kg) was extracted twice with MeOH (3 L, 6 h) by refluxing in a heating mantle. The crude extract (150 g) was suspended in distilled water (DW), and then solvent partitioned using n-Hexane, EtOAc, and *n*-BuOH. The EtOAc fractions (4.9 g) were divided into 14 fractions through the MPLC system (puriFlash^TM^430, interchim, Los Angeles, CA, USA, silica gel 230–400 mesh, 500 g, 20 mL/min, CHCl_3_:MeOH = 1:0~0:1). The 10 fraction was subjected to a prepLC (SpotⅡ, Armen, France, YMC SIL, 250 × 20 mm, 5 μM, 6 mL/min, CHCl_3_:MeOH:DW = 7:3:1 lower phase). The active compound (63.04 mg) was obtained from four subfractions (Figure 1A). The structure of calycosin-7-O-β-glucoside was identified by comparing it to the spectral data in [12]. The ^1^H and ^13^C nuclear magnetic resonance (NMR) spectra, high-performance liquid chromatography (HPLC) chromatogram, and chemical structure of Caly (>95% purity) are shown in Figure 1B–D. Caly was treated in MSCs for 24 h to evaluate cell toxicity at concentrations ranging from 0.1 to 30 μM using a 3-[4,5-dimethylthiazol-2-yl]-2,5-diphenyltetrazolium bromide (MTT) assay. The measurements showed that the concentrations of Caly did not influence cytotoxicity against MSCs (Figure 1E). 

### 2.2. Caly Enhances the Cell Migration, ALP Staining and Activity, and ARS Staining during the Osteogenesis of Human MSCs

**Figure 2:**
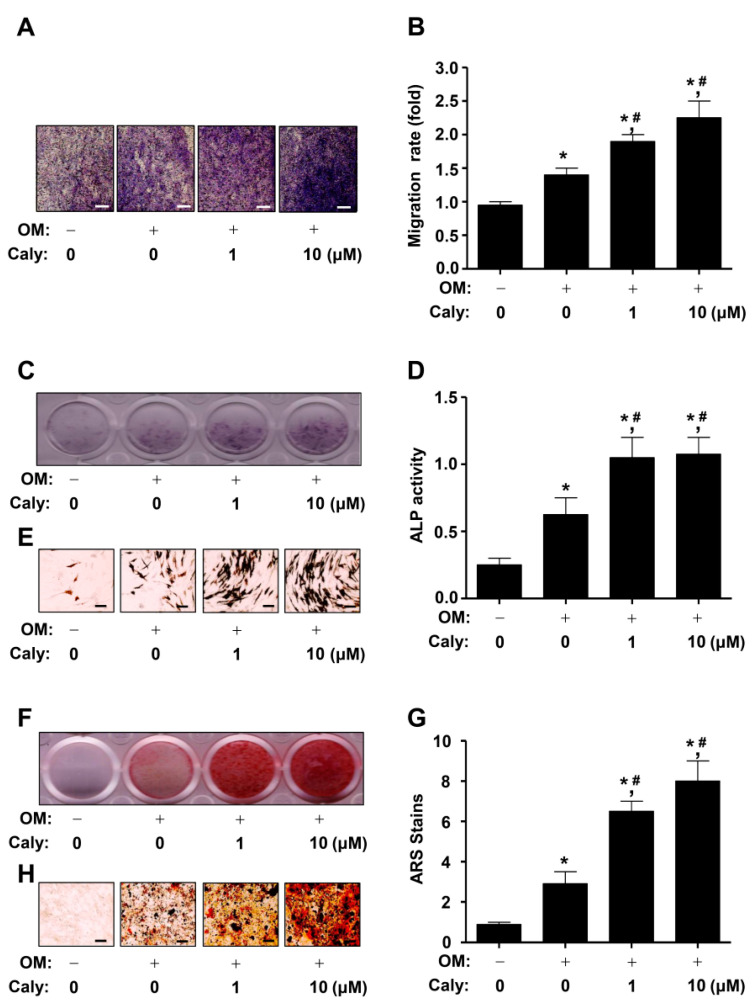
Effects of Caly on the osteogenesis of human MSCs. (**A**) Cell migration was measured by the Boyden chamber assay. The migrated cells were stained with 0.1% crystal violet and visualized under a light microscope. Scale bar: 50 μM. (**B**) The bar graph shows cell migration rate (fold) normalized to the control. (**C**–**E**) Early osteogenesis was measured by the ALP staining and activity assay at 7 days. The early osteogenic cells were stained with ALP reaction solution. The staining was visualized under a scanner (**C**). ALP activity was quantitatively measured using a spectrophotometer (**D**). ALP-positive expressing cells were visualized under a light microscope (**E**). Scale bar: 50 μM. (**F**–**H**) Late osteogenesis was measured by ARS staining assay at 21 days. After staining with 2% ARS solution, the mineralization was visualized under a scanner (**F**), quantitatively analyzed using a spectrophotometer (**G**), and visualized under a light microscope (**H**). Scale bar: 100 μm. Data are expressed as the mean ± S.E.M. from three separate experiments (* *p* < 0.05 compared to the control, and # *p* < 0.05 compared to OM).

The osteogenesis of MSCs and osteoblast lineages are initiated by the migration to bone formation and repair sites. To determine whether Caly influences cell migration, we caused the osteogenic differentiation of human MSCs using osteogenic supplement medium (OM) with or without Caly for 24 h, after which the migration was evaluated using a Boyden chamber assay. The assay exhibited that 1–10 μM Caly significantly promoted the mobility, compared to OM alone (Figure 2A,B). Next, to examine the biological effects of Caly in early osteogenesis, we measured the effects on the osteogenic differentiation of Caly using alkaline phosphatase (ALP) staining, which is used as an early phase marker during osteogenesis, after cultivating MSCs in OM with or without Caly for 7 days. The results exhibited that 1–10 μM Caly elevated the staining of ALP (Figure 2C). Subsequently, we carried out an ALP activity assay in response to 1–10 μM Caly treatment using a spectrophotometer. The results exhibited that 1–10 μM Caly significantly elevated the activity of ALP (Figure 2D). The ALP-positive expressing cells were confirmed using a light microscope (Figure 2E). Mineralized nodule formation was further evaluated using Alizarin red S (ARS) staining to monitor the degree of matrix mineralization, which is used as a late phase marker during osteogenesis, after MSCs were cultivated in OM with or without Caly for 21 days. The mineralization was detected using a scanner, and the results exhibited that 1–10 μM Caly promotes the late osteogenesis of MSCs (Figure 2F). Quantitatively, the Caly-stimulated late osteogenesis was validated using a spectrophotometer (Figure 2G). In addition, the mineralized nodules were observed using a light microscope (Figure 2H).

### 2.3. Caly Activates the BMP2, Wnt3a, and AKT Signaling Pathways during the Osteogenesis of Human MSCs

**Figure 3:**
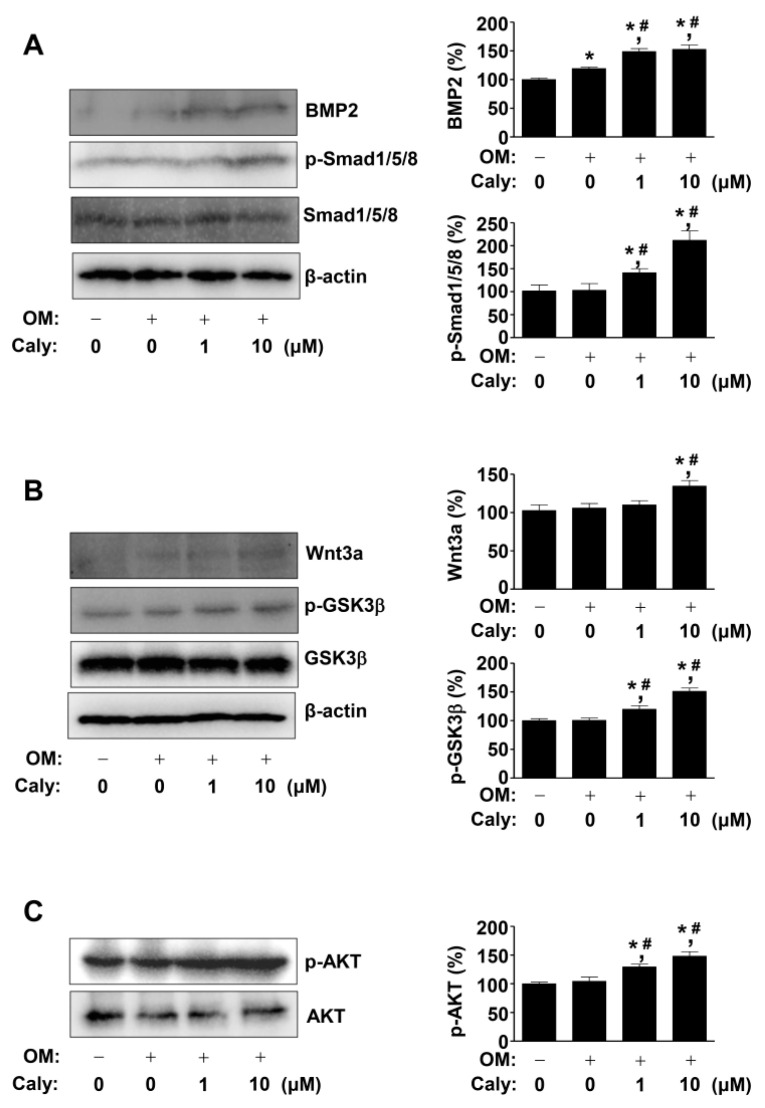
Effects of Caly on the BMP2, Wnt3a, and AKT signaling pathways during the osteogenesis of human MSCs. (**A**–**C**) BMP2 expression, Smad1/5/8 phosphorylation (p-Smad1/5/8), β-actin expression (**A**), Wnt3a expression, GSK3β phosphorylation (p-GSK3β), β-actin expression (**B**), AKT phosphorylation (p-AKT), and AKT expression (**C**) were analyzed using Western blot analysis. The bar graph shows relative expression level (%) normalized to the control (right). Data are expressed as the mean ± S.E.M. from three separate experiments (* *p* < 0.05 compared to the control, and # *p* < 0.05 compared to OM).

To elucidate the signaling pathways associated with the Caly-stimulated osteogenesis in MSCs, BMP2, Wnt3a, and AKT were evaluated by Western blot analysis to detect the levels of protein expression and phosphorylation. The results showed that first, 1–10 μM Caly enhanced BMP2 expression and Smad1/5/8 phosphorylation, compared to that with OM alone (Figure 3A). Second, 1–10 μM Caly enhanced Wnt3a expression and GSK3β phosphorylation, compared to that with OM alone (Figure 3B). Third, 1–10 μM Caly enhanced AKT phosphorylation, compared to that with OM alone (Figure 3C). These data imply that Caly will regulate RUNX2, which is a key protein for the osteogenesis of MSCs.

### 2.4. Caly-Activated Signaling Increases RUNX2 Expression and Promotes the Osteogenesis of Human MSCs

**Figure 4:**
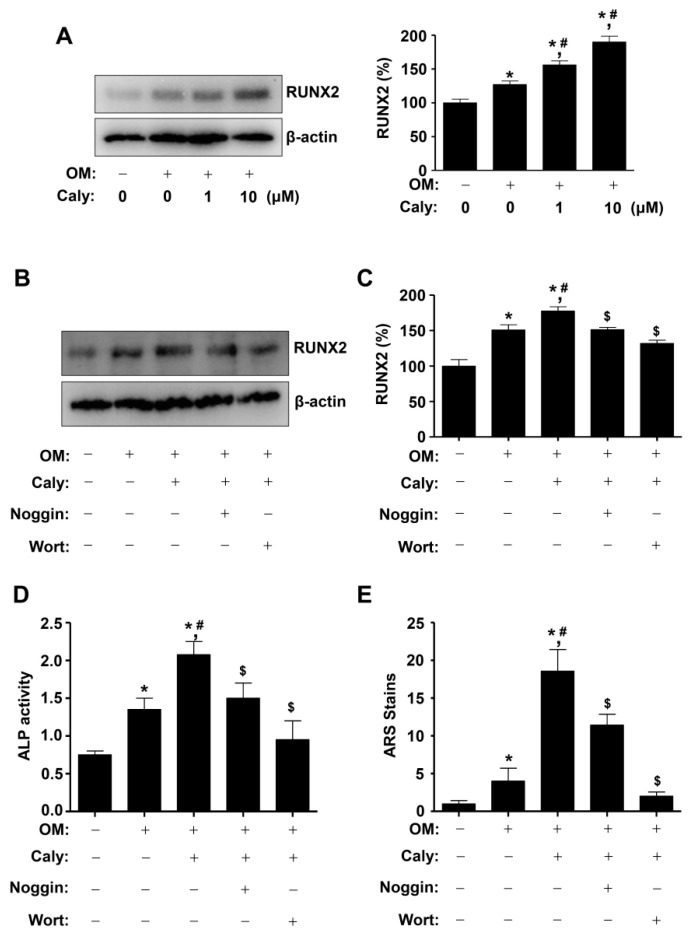
Caly potentiates RUNX2 expression during the osteogenesis of human MSCs. (**A**) RUNX2 and β-actin expressions were analyzed using Western blot analysis at 2 days. The bar graph shows relative RUNX2 level (%) normalized to the control (right). (**B**,**C**) After the pretreatment of Noggin and Wort, RUNX2 and β-actin expressions were analyzed using Western blot analysis at 2 days (**B**) The bar graph shows relative RUNX2 level (%) normalized to the control (**C**). (**D**,**E**) After the pretreatment of Noggin and Wort, ALP activity at 7 days (**D**) and ARS stains at 21 days (**E**) were quantitatively measured using a spectrophotometer. Data are expressed as the mean ± S.E.M. from three separate experiments (* *p* < 0.05 compared to the control, # *p* < 0.05 compared to OM, and $ *p* < 0.05 compared to OM + Caly).

Since RUNX2 is a main convergence protein of the BMP2, Wnt3a, and AKT signaling pathways that cause the osteogenesis of human MSCs, we clarified whether Caly influenced RUNX2 expression. As shown in Figure 4A, 1–10 μM Caly enhanced the expression of RUNX2, compared to that with OM alone (Figure 4A). To demonstrate the functional association of the expression of RUNX2 with Caly-activated signaling, MSCs were incubated with 10 μM Caly in the pretreatment of Noggin, a BMP2 inhibitor, and Wortmannin (Wort), an AKT inhibitor. The increased expression of RUNX2 was significantly inhibited by Noggin and Wort during the osteogenesis of MSCs (Figure 4B,C). Moreover, we demonstrated that Noggin and Wort significantly abolished Caly-stimulated ALP activity and mineralized nodule formation during the early and late osteogenesis of MSC (Figure 4D,E).

### 2.5. Caly Promotes RUNX2 Expression and Osteogenesis in Pre-Osteoblasts Committed to the Osteoblast Lineages from MSCs

In addition, we investigated whether Caly influences osteogenesis in pre-osteoblasts committed to the osteoblast lineages from MSCs. The migration was carried out after inducing osteoblast differentiation using osteogenic supplement medium (OS) with or without Caly for 24 and 48 h. The migration assay exhibited that 1–10 μM Caly significantly enhanced the mobility at 24 h, compared to OS alone (Supplementary Figure S1A,B). As shown in Supplementary Figure S1C,D, 1–10 μM Caly significantly increased osteogenesis in pre-osteoblasts (Supplementary Figure S1C,D). In addition, Western blot analysis and immunofluorescence observation exhibited that 1–10 μM Caly increased the total and nuclear RUNX2 expression during osteogenesis in pre-osteoblasts (Supplementary Figure S2A,B), suggesting that Caly also stimulates RUNX2 expression and osteogenesis in pre-osteoblasts committed to the osteoblast lineages from MSCs. 

## 3. Discussion

Bone is continuously regenerated by bone cells including osteoblast lineages, which maintain a healthy skeleton throughout life [13,14]. MSCs are differentiated into osteoblast lineages to regulate bone development, regeneration, and repair processes through bone protein synthesis and matrix mineralization [14,15]. Abnormalities of the physiological process cause bone diseases, including osteoporosis and periodontitis [16,17,18]. As anabolic drugs, compounds isolated from plants have been investigated to prevent and cure bone diseases [19,20,21]. Previously, our group demonstrated the beneficial effects of various natural compounds from plants on osteoblast lineages [22,23,24,25,26,27]. In the present study, we demonstrated the osteogenic function of Caly obtained from *A. membranaceus* in human MSCs. 

The differentiation of MSCs is caused by complex processes including cell migration, proliferation, commitment to osteoblast lineages, and the maturation and mineralization of osteoblast leading to bone formation and repair in target areas [28,29]. Our present results demonstrated that Caly potentiates the cell migration, the activity and expression of ALP, and the formation of mineralized nodules by osteoblast lineages differentiated from MSCs. The recruitment of MSCs from various locations including the bone marrow, periosteum, and circulating blood to target areas is necessary during bone formation and repair [28,29,30]. It was reported that ALP expression and activity are elevated in osteoblast lineages differentiated from MSCs, and the calcium deposition of the organic bone matrix is increased during osteoblast maturation [31,32,33,34,35,36]. Therefore, the findings suggest that Caly has biological effects on the migration of MSCs, and their commitment to the osteoblast lineage, and the maturation of osteoblasts, triggering the processes of bone formation and repair.

The BMP2, Wnt3a, and AKT signaling pathways are involved in osteogenesis and bone formation [37,38,39,40]. The interaction of BMP2 with BMP receptors stimulates the phosphorylation of Smad1/5/8 proteins. The phosphorylated Smad1/5/8 proteins form complexes with Smad4, translocate from cytosol into nucleus, and then cause gene transcription [41]. The interaction of Wnt3a with Frizzled and LRP5/6 receptors increases the phosphorylation of GSK3β protein, inhibits the activities of GSK3β that lead to β-catenin degradation, and then causes gene transcription in the nucleus [42]. In addition, BMP2 and Wnt3a stimulated AKT serine/threonine kinase activity in a PI 3-kinase-dependent manner, respectively, and AKT signaling promotes osteogenesis [43,44,45]. Previous papers have also demonstrated osteogenic effects in rat calvarial osteoblasts [46,47]. Consistent with the previous literature, our present results demonstrated that Caly stimulates the BMP2, Wnt3a, and AKT signaling pathways for osteogenic differentiation and bone-forming activities. It was reported that these signaling pathways consequently modulate RUNX2 expression and are functionally integrated for osteogenesis [48,49,50,51]. In vivo and in vitro studies demonstrated that RUNX2 is a core transcription factor for bone-specific gene expression and matrix mineralization during osteogenesis and bone formation [52]. Moreover, in the present study, we validated the osteogenic effects of Caly on pre-osteoblasts committed to the osteoblast lineages from MSCs. These findings suggest that the functional cross-talks of Caly stimulated these signaling pathways to induce osteogenesis and bone formation through RUNX2 expression in MSCs and osteoblast lineages.

In conclusion, the safety and efficiency of natural compounds have received attention in various diseases, including bone diseases [53,54,55,56,57]. Diet is also important in preventing the risk of osteoporosis and treating patients with osteoporosis [58,59,60]. In particular, isoflavone-rich cowpea has been reported to increase the proliferation and differentiation of human osteoblasts through the activation of the BMP2 pathway. Soybean isoflavone treatment has been reported to induce osteoblast differentiation and proliferation through the activation of the Wnt3 pathway [61]. It has been reported that Icariin exhibits estrogen-like properties to induce osteogenic effects through the activation of the AKT pathway [62]. In the present study, we demonstrated that Caly induces RUNX2 expression by regulating the BMP2, Wnt3a/GSK, and AKT pathways to cause osteogenesis in human MSCs. Previously, relevant papers have also demonstrated that Caly has osteogenic effects in mouse stromal ST2 cell lines and rat calvarial osteoblasts [46,47,63]. Based on the previous literature and our data, our findings suggest that Caly may be developed for the prevention and treatment of bone diseases as an anabolic agent or used by being incorporated into adequate dietary intake to stimulate the osteogenesis of MSCs.

## 4. Materials and Methods

### 4.1. General and Plant Materials

Nuclear magnetic resonance (NMR) spectra were obtained using a JEOL ECX-500 spectrometer (JEOL Ltd., Tokyo, Japan) operating at ^1^H-NMR (500 MHz) and ^13^C-NMR (125 MHz) with tetramethylsilane (TMS) as internal standard. High-performance liquid chromatography (HPLC) was performed using Agilent 1260 series (Agilent Technologies, Santa Clara, CA, USA). The MPLC system (puriFlash^TM^430, interchim, Los Angeles, CA, USA) and prep-LC equipment (SpotⅡ, Armen, France) and Silica gel 60 (230–400 mesh ASTM, Merck, Darmstadt, Germany) were used to separate the active compound. The *A. membranaceus* were purchased at a commercial herbal medicine market. A voucher specimen (P357) has been deposited in the Natural Products Bank, National Institute for Korean Medicine Development (NIKOM).

### 4.2. Calycosin-7-O-β-glucoside (Caly)

White powder, EI-MS *m*/*z* 446.40 [M]^+^, molecular formula C_22_H_22_O_10_; ^1^H-NMR (500 MHz, DMSO-d_6_) δ 3.27–3.34 (2H, m, H-2″, 3″), 3.16 (1H, t, *J* = 9.0 Hz, H-4″), 3.44 (1H, m, H-5″), 3.48 (1H, dd, *J* = 5.5, 11.5 Hz, H-6″a), 3.71 (1H, dd, *J* = 4.5, 11.5 Hz, H-6″b), 3.79 (3H, s, OCH_3_), 4.60 (1H, t, *J* = 6.0 Hz, 6″-OH), 5.08 (1H, t, *J* = 5.1 Hz, 4″-OH), 5.09 (1H, d, *J* = 7.8 Hz, H-1″), 5.14 (1H, d, *J* = 4.5 Hz, 3″-OH), 5.43 (1H, d, *J* = 4.8 Hz, 2″-OH), 6.96 (2H, br s, H-5′, 6′), 7.06 (1H, s, H-2′), 7.13 (1H, dd, *J* = 2.4, 8.7 Hz, H-6), 7.22 (1H, d, *J* = 2.4 Hz, H-8), 8.04 (1H, d, *J* = 8.7 Hz, H-5), 8.39 (1H, s, H-2), 9.02 (1H, s, 3-OH); ^13^C-NMR (125 MHz, DMSO-d_6_) δ 153.6 (C-2), 124.4 (C-3), 174.6 (C-4), 127.0 (C-5), 115.6 (C-6), 161.4 (C-7), 103.4 (C-8), 157.0 (C-9), 118.5 (C-10), 123.6 (C-1′), 116.4 (C-2′), 146.0 (C-3′), 147.6 (C-4′), 111.9 (C-5′), 119.7 (C-6′), 55.7 (OCH_3_), 100.0 (C-1″), 73.1 (C-2″), 76.5 (C-3″), 69.6 (C-4″), 77.2 (C-5″), 60.6 (C-6″). Caly powder was dissolved in 100% DMSO, and the stock was diluted at 1:1000. A final concentration of 0.1% DMSO was used as the control.

### 4.3. Human Mesenchymal Stem Cell (MSC) Culture

Human bone marrow-derived MSCs were purchased from ScienCell Research Laboratories (Carlsbad, CA, USA) and cultured in Mesenchymal Stem Cell Medium (Carlsbad, CA, USA) containing basal medium, fetal bovine serum (FBS), mesenchymal stem cell growth supplement, and penicillin/streptomycin solution. MSCs were then maintained at 37 °C in a humidified atmosphere with 5% CO_2_. 

### 4.4. Pre-Osteoblast Culture

Pre-osteoblast MC3T3-E1 cells were purchased from the American Type Culture Collection (ATCC, CRL-2593) (Manassas, VA, USA) and cultured in α-minimum essential medium (WELGEME, Inc., Seoul, Republic of Korea) without L-AA containing 10% FBS and 1 X Gibco^®^ Antibiotic-Antimycotic (Thermo Fisher Scientific, Waltham, MA, USA) at 37 °C in a humidified atmosphere of 5% CO_2_ and 95% air, as previously described [64].

### 4.5. Osteogenesis of MSCs and Pre-Osteoblasts

The osteogenesis of MSCs was induced by changing to OM containing 10 mM β-glycerophosphate (β-GP), 50 μg/mL L-ascorbic acid (L-AA), and 10 nM dexamethasone (DEX) (Sigma-Aldrich, St. Louis, MO, USA). The medium was replaced every 2 days during the incubation period. 

The osteogenesis of pre-osteoblasts was induced using OS containing 10 mM β-GP and 50 μg/mL L-AA. The medium was replaced every 2 days during the incubation period, as previously described [64]. 

### 4.6. Cell Viability Assay

Cells (1 × 10^4^ cells/well) were seeded onto 96-well plates, and cell viability was measured by performing an MTT assay to detect NADH-dependent dehydrogenase activity retained in living cells as previously described [64]. Briefly, cells were incubated with MTT solution for 2 h, and formazan was solubilized using 100% DMSO. The absorbance was monitored at 540 nm using the Multiskan GO Microplate Spectrophotometer (Thermo Fisher Scientific).

### 4.7. Migration Assays

Boyden chamber assay and wound-healing assay were performed as previously described [64]. Briefly, for Boyden chamber assay, a nucleopore filter was coated with Matrigel, and the 2 × 10^4^ cells were incubated in the Boyden chamber for 4 h at 37 °C in a humidified atmosphere of 5% CO_2_ and 95% air. Then, the cells were fixed and stained with 0.5% crystal violet. For wound-healing assay, cells (1 × 10^6^ cells/well) were seeded, wounded, and incubated for 24 h at 37 °C in a humidified atmosphere of 5% CO_2_ and 95% air. Cell migration was visualized using light microscopy.

### 4.8. ALP Staining Assay and ALP Activity Assay

Osteogenesis was induced for 7 days, and the assays were performed as previously described [64]. Briefly, for ALP staining assay, cells (2 × 10^4^ cells/well) were seeded, fixed, and incubated for 1 h at 37 °C with ALP reaction solution (Takara Bio Inc., Tokyo, Japan). The level of ALP staining was observed using a scanner and colorimetric detector (ProteinSimple Inc., Santa Clara, CA, USA). ALP activity assay was carried out using an alkaline phosphatase activity colorimetric assay kit, and the activity was quantitatively monitored at 405 nm using the Multiskan GO Microplate Spectrophotometer (Thermo Fisher Scientific).

### 4.9. ARS Staining Assay

The osteogenesis of MSCs was induced for 21 days, and the assay was performed as previously described [64]. Briefly, cells (2 × 10^4^ cells/well) were seeded, fixed, and stained with 2% Alizarin red S (pH 4.2) (Sigma-Aldrich) for 10 min. For quantification, stains were dissolved in 100% DMSO, and the absorbance was monitored at 590 nm using the Multiskan GO Microplate Spectrophotometer (Thermo Fisher Scientific).

### 4.10. Western Blot Analysis

Western blot analysis was performed as previously described [65]. Briefly, proteins were transferred to polyvinylidene difluoride membranes (Millipore, Bedford, MA, USA), and the membranes were blocked for 1 h at room temperature and incubated overnight at 4 °C with the primary antibodies as follows: AKT (1:1000, #4691, Cell Signaling Technology, Beverly, MA, USA), p-AKT (1:1000, #4060, Cell Signaling Technology), β−actin (1:1000, #sc-47778, Santa Cruz Biotechnology, Santa Cruz, CA, USA), BMP2 (CUSABIO, #CSB-PAO9419AORb, Houston, TX, USA), Smad1/5/8 (1 : 1000, #sc-6031-R, Santa Cruz Biotechnology), p-Smad1/5/8 (1:2000, 13820, Cell Signaling), GSK3β (1:1000, #12456, Cell Signaling), p-GSK3β (1:1000, #9336, Cell Signaling), RUNX2 (1:1000, #12556, Cell Signaling Technology), and Wnt3a (1:1000, #2721, Cell Signaling). Then, the membrane was incubated with horseradish peroxidase-conjugated secondary antibodies (1:10,000, Jackson ImmunoResearch, West Grove, PA, USA) for 1 h at room temperature. The protein signals were monitored in the ProteinSimple detection system (ProteinSimple Inc., Santa Clara, CA, USA).

### 4.11. Immunofluorescence

Immunofluorescence assay was performed as previously described [64]. Briefly, cells (1 × 10^4^ cells/well) were seeded onto 8-well chamber slides (Thermo Fisher Scientific), fixed with 10% formalin for 10 min at room temperature, permeabilized with 0.2% Triton X-100 for 20 min, and blocked with 3% BSA for 1 h. The cells were incubated with anti-RUNX2 antibody (1:200, Cell Signaling Technology) overnight at 4 °C, followed by incubation with Alexa Fluor 568-conjugated secondary antibody (1:500, Invitrogen, Carlsbad, CA, USA) for 2 h and stained with DAPI (Sigma-Aldrich, St. Louis, MO, USA) for 10 min at room temperature. The slides were washed three times and mounted using Fluoromount^TM^ Aqueous Mounting Medium (Sigma-Aldrich).

### 4.12. Statistical Analysis

Data were analyzed using an unpaired Student’s t-test in the GraphPad Prism Version 5 program (GraphPad Software, Inc., San Diego, CA, USA). A value of *p* < 0.05 was considered to be statistically significant.

## Data Availability

Data are contained within the article or supplementary material.

## Supplementary Materials

The following are available online at https://www.mdpi.com/article/10.3390/ijms222111362/s1.

## Abbreviations

ALP	Alkaline phosphatase
ARS	Alizarin red S
β-GP	β-glycerophosphate
BMP	Bone morphogenetic protein
Caly	Calycosin-7-O-β-glucoside
L-AA	L-ascorbic acid
MSCs	Mesenchymal stem cells
MTT	3-[4,5-dimethylthiazol-2-yl]-2,5-diphenyltetrazolium bromide
OM	Osteogenic supplement medium for the osteogenesis of MSCs containing 10 mM β-glycerophosphate (β-GP), 50 μg/mL L-ascorbic acid (L-AA), and 10 nM dexamethasone (DEX)
OS	Osteogenic supplement medium for the osteogenesis of pre-osteoblasts containing 10 mM β-glycerophosphate (β-GP) and 50 μg/mL L-ascorbic acid (L-AA)
RUNX2	Runt-related transcription factor 2
