# Supplementary material for: Calycosin-7-O-β-Glucoside Isolated from Astragalus membranaceus Promotes Osteogenesis and Mineralization in Human Mesenchymal Stem Cells
Source: Int J Mol Sci. 2021 Oct 21;22(21):11362. doi: 10.3390/ijms222111362 (PMC8583672; doi:10.3390/ijms222111362)

Supplementary Figure Legends

Supplementary Figure 1. Effects of Caly on the osteogeneis of pre-osteoblasts committed to the osteoblast lineages. (**A**) Cell migration was measured by the Wound healing assay. The migrated cells were visualized under a light microscope. Scale bar: 100 μm. **(B)** The line graph shows migration rate (%) normalized to the control. **(C)** The ALP staining was observed at 7 days using a scanner (upper) and colorimetric detector (middle). ALP-positively expressing cells were visualized under a light microscope (bottom). Scale bar: 50 μm. **(D)** ALP activity was quantitatively measured at 7 days using a spectrophotometer. Data are expressed as the mean ± S.E.M. from three separate experiments (*$p < 0.05$ compared to the control, and # $p < 0.05$ compared to OS).

Supplementary Figure 2. Effects of Caly on the RUNX2 expression of pre-osteoblasts committed to the osteoblast lineages. (**A**) RUNX2 and β-actin expressions were analyzed using western blot analysis at 2 days. The bar graph shows relative RUNX2 level (%) normalized to the control (left). **(B)** RUNX2 expression in nucleus was measured at 2 days using immunofluorescence. RUNX2 (red color), nuclear marker DAPI (blue color), Merged (purple color). Scale bar: 50 μm. Data are expressed as the mean ± S.E.M. from three separate experiments (*$p < 0.05$ compared to the control, and # $p < 0.05$ compared to OS).

Supplementary Figure 1

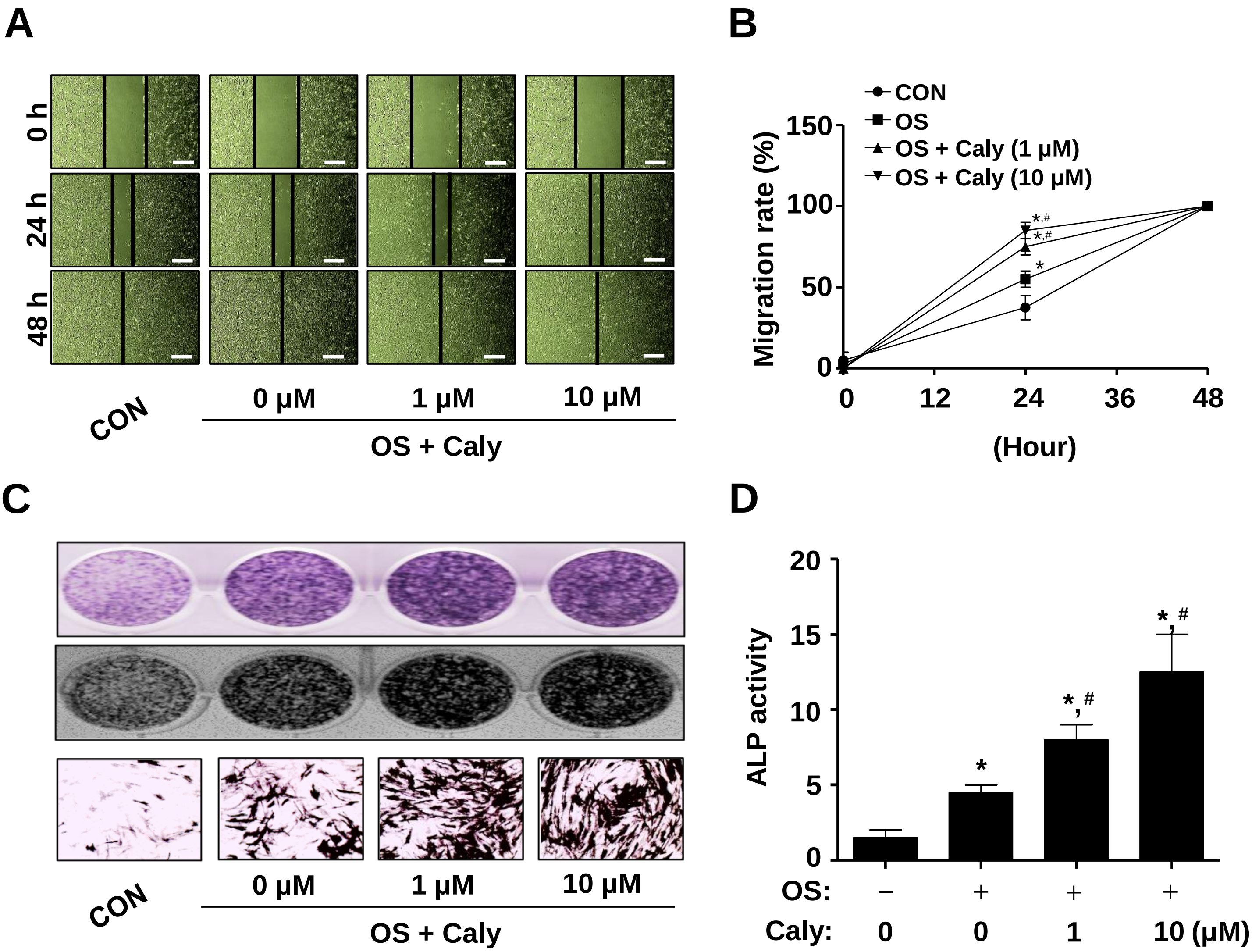

Supplementary Figure 2

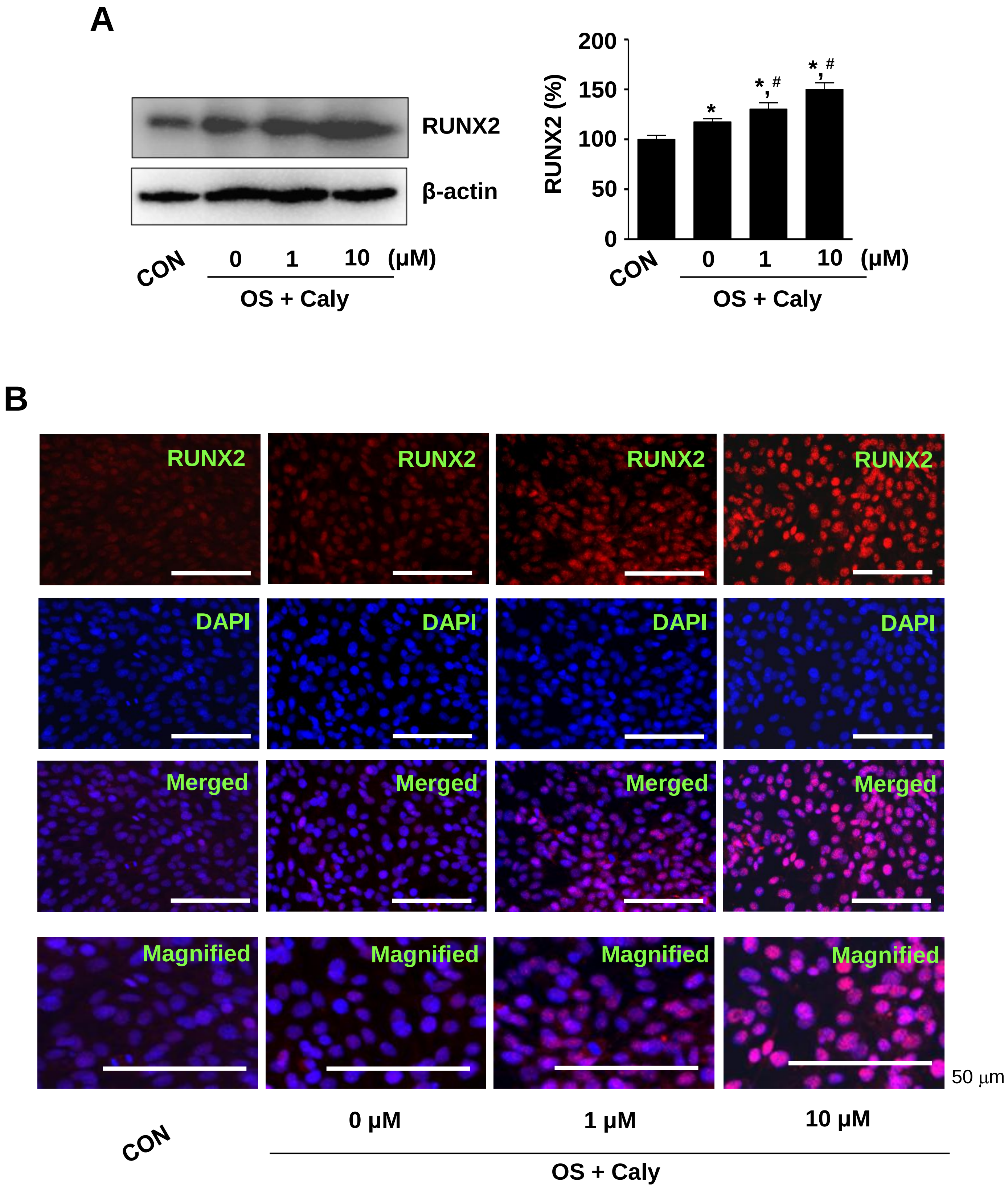

Supplement: Supplementary file 1 [file ijms-22-11362-s001.zip › ijms-1408307-supplementary.pdf]
